# Hydroxide Mobility in Aqueous Systems: Combining *Ab Initio* Accuracy with Millisecond Timescales

**DOI:** 10.1002/smll.202500931

**Published:** 2025-07-17

**Authors:** Jonas Hänseroth, Daniel Sebastiani, Johnny Alexander Jimenez Siegert, Jakob Scholl, Karl Skadell, Christian Dreßler

**Affiliations:** ^1^ Department of Theoretical Solid State Physics, Institute of Physics Technische Universität Ilmenau 98693 Ilmenau Germany; ^2^ Theoretical Chemistry Martin‐Luther Universität Halle‐Wittenberg 06120 Halle (Saale) Germany; ^3^ Fraunhofer‐Institut für Keramische Technologien und Systeme IKTS Hydrogen Technologies 99310 Arnstadt Germany

**Keywords:** anion‐exchange membrane water electrolysis, hydroxide ion dynamics, molecular dynamics simulations, multiscale simulations, proton transport

## Abstract

A multiscale simulation approach is presented for hydroxide transport in aqueous solutions of potassium hydroxide, combining *ab initio* molecular dynamics (AIMD) simulations with force field ensemble averaging and lattice Monte Carlo techniques. This method achieves near *ab initio* accuracy by capturing the femtosecond scale dielectric relaxation dynamics of the aqueous hydrogen bonding network, while extending the simulation capability to millisecond diffusion timescales. This extraordinary extension of the available length and time scales enables future studies of hydroxide mobility in functional materials such as nanostructured anion‐exchange membranes, where hydroxide ions migrate through nanometer‐sized channels. Remarkably, this approach demonstrates that a single AIMD trajectory is sufficient to predict hydroxide conductivity over a range of concentrations, underscoring its computational efficiency and relevance to the design of advanced energy materials.

## Graphical abstract

A multiscale simulation strategy for hydroxide transport in aqueous potassium hydroxide is presented, integrating ab initio molecular dynamics with force field and Monte Carlo methods. The approach links femtosecond‐scale hydrogen‐bond dynamics to millisecond‐scale diffusion while maintaining near‐ab initio accuracy. It enables atomistic insight into hydroxide mobility in complex nanostructured environments.

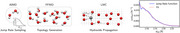

## 1 Introduction

Green hydrogen, produced by the electrolysis of water using renewable electricity, plays a crucial role in the global transition to a more sustainable energy economy.^[^ ^1^ ^]^ Among the various methods for water splitting, anion‐exchange membrane (AEM) water electrolysis stands out due to its high efficiency and its ability to utilize inexpensive and abundant electrode materials, such as iron and nickel.^[^ ^2^, ^3^, ^4^, ^5^, ^6^ ^]^ In contrast, proton exchange membrane (PEM) electrolysis operates under acidic conditions and relies on noble metal catalysts like platinum and iridium, which are scarce and costly.^[^ ^7^, ^8^, ^9^ ^]^

Two key challenges for advancing AEM technology are improving membrane stability under alkaline operating conditions and enhancing hydroxide conductivity.^[^ ^3^, ^10^ ^]^ Achieving the latter would benefit greatly from simulation tools capable of predicting hydroxide mobility with low computational effort. Such tools would enable the optimization of AEM materials before synthesis, accelerating their development. However, simulating the complex polymeric systems with solvated nanochannels, as found in AEMs, requires large‐scale supercomputers and remains beyond the reach of state‐of‐the‐art molecular dynamics (MD) simulations. Current simulations of hydroxide dynamics are often limited to small model systems, where the polymeric structure of the membrane is replaced by small organic molecules that mimic its functional groups.^[^ ^11^, ^12^, ^13^, ^14^, ^15^, ^16^, ^17^ ^]^

While force field molecular dynamics (FFMD) simulations are computationally less demanding, they are inadequate for modeling hydroxide mobility.^[^ ^18^ ^]^ This is because hydroxide ion transport involves bond‐breaking and bond‐forming events, which can only be captured accurately using quantum chemical methods.^[^ ^13^, ^19^, ^20^ ^]^

To address this computational bottleneck, two alternative approaches have been proposed. The first one employs machine‐learning‐based interatomic force fields, which can simulate hydroxide ion mobility on timescales almost comparable to classical MD simulations while retaining near *ab initio* accuracy.^[^ ^21^, ^22^, ^23^, ^24^ ^]^ The second approach combines MD simulations with other methods, such as Monte Carlo simulations, to model ion dynamics on much larger timescales, extending up to milliseconds. By applying the MD–Monte Carlo technique to simulate ion transfer in the system, the determination of OH^−^ diffusion coefficients remains physically interpretable, rather than relying on the black‐box nature of machine‐learning‐based interatomic potentials. Moreover, this approach is faster than classical force field molecular dynamics, while machine‐learning molecular dynamics are comparatively slower, making the LMC method particularly well‐suited for investigating ion dynamics over extended timescales and within complex environments.

In this article, we focus on the second approach, adapting our previously developed combined Molecular Dynamics/Lattice Monte Carlo (cMD/LMC) framework for simulating proton dynamics to hydroxide ions.^[^ ^25^, ^26^, ^27^, ^28^ ^]^ This adaptation is motivated by the mechanistic similarities and differences between the conduction of hydronium ions (H_3_O^+^) and hydroxide ions (OH^−^). While H_3_O^+^ forms via the addition of a proton to a water molecule, OH^−^ forms through proton removal. Both exhibit enhanced mobility compared to water molecules, attributed to the Grotthuss mechanism, which facilitates ion transport through a combination of hopping and reorientation steps.^[^ ^19^, ^29^, ^30^, ^31^ ^]^

The Grotthuss mechanism involves proton transfer through a complex three‐dimensional hydrogen bond network of water molecules. The process includes a hopping step, where a proton is transferred within the hydrogen bond network, followed by a reorientation of water molecules to facilitate further transfers. Due to the Grotthuss mechanism, increased mobility can be observed for both ion types, with protons exhibiting greater mobility than hydroxide ions, as reflected in their diffusion coefficients (experimental ratio: D(H^+^) / D(OH^−^) ≈ 2, for both regular and deuterated species).^[^ ^19^, ^32^, ^33^ ^]^ This factor arises from distinct conduction mechanisms between protons and hydroxide ions.

The “proton‐hole mechanism” suggests a one‐to‐one correspondence between proton and hydroxide conduction, involving a threefold coordinated OH^−^ and an intermediate $H_{3}O_{2}^{-}$ complex analogous to H_3_O^+^(H_2_O)_3_ and $H_{5}O_{2}^{+}$.^[^ ^34^ ^]^ However, *ab initio* studies by Tuckerman et al. demonstrate a distinct hydroxide transfer mechanism.^[^ ^35^ ^]^ The initial step involves a transition from a square‐planar coordinated hydroxide ion (OH^−^(H_2_O)_4_) to a tetrahedral geometry (OH^−^(H_2_O)_3_). Notably, the Zundel‐analog complex ($H_{3}O_{2}^{-}$) exists only transiently, persisting for just 2–3 oscillation periods during the transfer mechanism.^[^ ^36^ ^]^ This behavior, observed through time‐resolved IR experiments, aligns with the “presolvation concept”, which emphasizes hypercoordination and dynamic solvation shell changes.^[^ ^19^, ^37^, ^38^ ^]^

In this work, we evaluate our multiscale approach for simulating hydroxide transport in aqueous potassium hydroxide solutions. We demonstrate that a single *ab initio* molecular dynamics trajectory within our framework is sufficient to predict hydroxide conductivity across a range of concentrations.[Supplementary-material smll202500931-supl-0001]

## 2 Method

**Figure 1:**
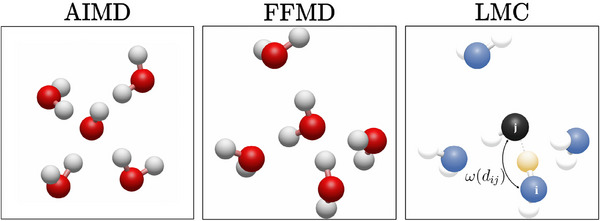
Local hydroxide transfer rates derived from an AIMD simulation of aqueous KOH and an oxygen lattice derived from an FFMD simulation of pure water are required for the cMD/LMC approach. The long‐range transport of hydroxide ions is calculated using these inputs in a Monte Carlo algorithm. The red atoms represent oxygen, while the white atoms correspond to hydrogen. Blue lattice sites denote water species, and the black lattice site represents a hydroxide species in the LMC approach. A proton undergoing a jump is highlighted in yellow, its probability of jumping from O_i_ to O_j_ is given by the term ω(*d*_*ij*_).

Our approach combines molecular dynamics simulations with a lattice Monte Carlo method to model hydroxide ion transport in aqueous solutions. The *ab initio* molecular dynamics (AIMD) simulations capture local hydroxide transfer rates on the sub‐picosecond timescale, while the Monte Carlo method simulates the long‐range propagation of hydroxide ions on an oxygen lattice derived from a force field molecular dynamics simulations of pure water. **Figure** **1** illustrates the approach.

Our aim is not to reproduce the diffusion coefficients of bulk hydroxide solution from AIMD trajectories, but rather to predict how realistic material inhomogeneities ‐ impurities, nanostructure, confinement, and other factors ‐ affect hydroxide transport. To achieve this, we have developed a statistical method that superimposes hydroxide dynamics onto classical water MD simulations of sizes that are infeasible for first‐principles treatment of OH^−^ ions. Because *ab initio* methods cannot handle such large systems directly, our approach propagates hydroxide motion on top of the oxygen atoms of the water molecules in the classical trajectories. It incorporates relevant inhomogeneities‐chemical impurities (e.g., gas bubbles), nanoscale features, interfaces, confinement effects, etc. ‐ by post‐processing a final MD trajectory. The result is an efficient, scalable estimate of hydroxide mobility in complex materials environments.

In the Monte Carlo approach, the system is simplified by representing only the oxygen atoms, which are either occupied by two protons (water molecules) or a single proton (hydroxide ions). The hydrogen atoms are fixed at the positions of their covalently bonded oxygen atoms, with the oxygen atom positions extracted from force field MD trajectories. While FFMD cannot simulate bond breaking or formation, our multiscale approach overcomes this limitation by enabling proton jumps between neighboring oxygen atoms. The probabilities of these proton jumps are determined using a jump rate function derived from quantum chemical simulations of solvated hydroxide ions.

This Monte Carlo method uniquely combines standard lattice‐based Monte Carlo and traditional kinetic Monte Carlo (KMC) approaches. In our method, the lattice is constructed using the oxygen positions from an underlying molecular dynamics trajectory, which are updated after each Monte Carlo step. Kinetic rates for proton transfer between neighboring lattice sites are periodically applied, with the fixed timestep determined by the time interval between consecutive frames in the MD trajectory.

### 2.1 Sampling Hydroxide Jump Rates From *Ab Initio* Molecular Dynamics Simulation

**Figure 2:**
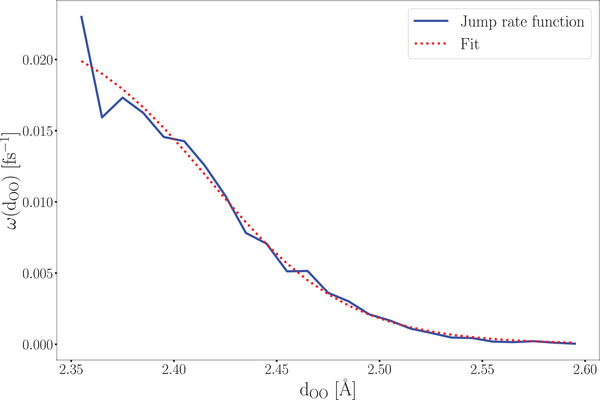
Jump probability for protons with respect to the O–O distance between the hydroxide ion and a neighboring water molecule. The jump rate function is sampled from the AIMD trajectory of aqueous KOH solution *c*(KOH) = 17.89 molL^−1^ at 333 K.

Previous studies have shown that proton transfer within the oxygen lattice is governed by the oxygen‐oxygen distances.^[^ ^25^, ^26^, ^27^, ^28^ ^]^ It turns out that the relationship between proton transfer probabilities and oxygen‐oxygen distances can be accurately described using a Fermi‐like function (refer to **Figure** **2** and Equation 1). The shape of this function is determined prior to the actual cMD/LMC runs on the basis of a comparably short *ab initio* molecular dynamics simulation. From this MD simulation, we compute the jump rate function ω(*d*_OO_) as the conditional probability for a jump at a given distance *d*_OO_ by counting the actual number of real jumps in the MD trajectory at this distance divided by the number of the overall occurrence of this oxygen‐oxygen distance between hydroxide ions and water molecules in the MD trajectory.

In our current realization, the function is derived from AIMD simulations that do not account for quantum effects. Much effort has already been put into the investigation of nuclear quantum effects on proton dynamics.^[^ ^39^, ^40^, ^41^, ^42^, ^43^ ^]^ Our approach offers flexibility to incorporate the correction from nuclear quantum effects on the jump rate function. The jump rate function could be sampled from *ab initio* path‐integral MD without modifying other components of the methodology.

For an accurate description of the resulting rate function ω(*d*_OO_) it is sufficient to have a modest number of proton jumps (per distance window) in the *ab initio* trajectory; it is not necessary to perform an extended *ab initio* simulation with a well‐converged proton diffusion statistics.

The numerically obtained conditional hopping probability is fitted to a Fermi‐Function according to Equation 1. The fit parameters for our system (aq. KOH solution at *c* = 17.89 molL^−1^ and at 333 K) are given in **Table** **1**.

(1) $$\omega \left(d_{ij}\right)=\frac{a}{1+\exp\left(\frac{d_{ij}-b}{c}\right)}$$

**Table 1:** Fermi fit parameters describing the jump rate function of the aqueous KOH solution with *c*(KOH) = 17.89 molL^−1^ at 333 K.

*a* [fs^−1^]	*b* [Å]	*c* [Å ^−1^]
0.023	2.4	30

Naturally, the jump probability ω(*d*_OO_) and thus its fit parameters (*a*, *b*, *c*) are concentration dependent. However we have explicitly checked that their variation within our concentration range is below 10 % for any parameter and does not exhibit a systematic trend. Thus, there variations are of the same amplitude as the statistical fluctuations in the numerically computed conditional hopping probabilities. Therefore, we have chosen to work with a single set if parameters *a*, *b*, *c* for all KOH concentrations.

### 2.2 Lattice Monte Carlo Algorithm

**Figure 3:**
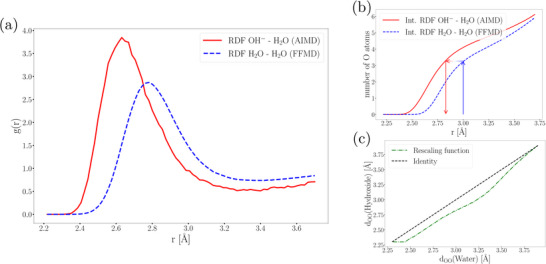
Distance rescaling process: a) Radial distribution function (RDF) of the OO distance for water and hydroxide ion, b) the integrated OO‐RDF, and c) the rescaling function, that maps water–water *d*_OO_ to hydroxide‐water *d*_OO_.

To perform our Lattice Monte Carlo (LMC) simulations, we use molecular‐dynamics trajectories of pure water that may include realistic material inhomogeneities ‐ chemical impurities (e.g., gas bubbles), nanostructural features, and confinement effects. This approach allows us to predict the dynamics of hydroxide moieties in complex‐system trajectories after the calculation of the trajectory, even when the original simulation box contains only water molecules without explicit OH^−^ ions or counter‐ions. The LMC algorithm extracts only oxygen atom positions from these trajectories, utilizing them as a dynamic oxygen atom lattice. Within our LMC framework, we designate a user‐defined subset of these oxygen atoms (which originated as water oxygens in the FFMD simulation) as hydroxide particles. However, this post‐simulation introduction of hydroxide ions presents a structural challenge: the environment around these designated hydroxide sites retains the characteristics of normal water molecules. This is problematic because the average O–O distance between a hydroxide ion and its nearest water molecule (2.6 Å) is measurably shorter than the average distance between two neutral water molecules (2.75 Å) (see **Figure** **3**a).

This discrepancy is evident in the integrated radial distribution functions (RDFs) shown in Figure 3b and poses a challenge for our proposed multiscale propagation scheme.

To apply the jump rates $\omega (d_{O_{\left({\mathrm{OH}}^{-}\right)}O_{\left(H_{2}O\right)}})$ to our pure water topology, we implement a distance transformation that rescales the separations between hydroxide‐occupied lattice sites and their neighboring sites. This rescaling function dynamically adjusts the local environment around hydroxide ions before each LMC time step. Our algorithm first measures the distances between a hydroxide site and nearby water sites, applies the rescaling function to these values, and then calculates jump rates using the corrected distances within our multiscale framework.

The fundamental purpose of this rescaling function is to account for the structural differences between hydroxide‐water and water‐water interactions. For example, while a water molecule typically has approximately 3.4 neighboring water molecules within a 3.0 Å radius, a hydroxide ion would have the same number of neighbors within a smaller radius of approximately 2.8 Å. The convex function maps distances from the range [2.25, 3.75] to the same range but redistributes values according to hydroxide‐water interaction patterns. In the example illustrated in Figure 3b, the function transforms a specific lattice site distance $d_{O_{i,({\mathrm{OH}}^{-})}O_{j,(H_{2}O)}}$ from 3.0 Å to 2.8 Å.

The rescaling function ensures that the local environment around a hydroxide‐occupied lattice site reflects the appropriate distances in the solvation structure observed in actual hydroxide‐water systems.

### 2.3 Simulation Scheme

**Figure 4:**
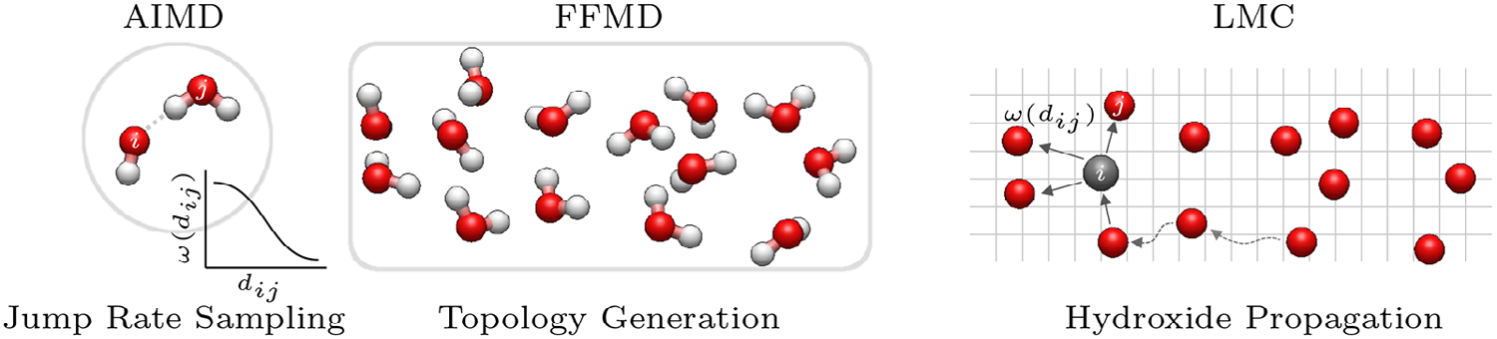
Combined Lattice Monte‐Carlo / Molecular Dynamics scheme: 1) A jump rate function is sampled from an AIMD trajectory; 2) An underlying topology is generated with a classical force field from a pure water trajectory; 3) The hydroxide moieties are propagated on top of the topology using the distance dependent jump probabilities while applying a rescaling procedure. The red spheres represent oxygen atoms and the white ones represent hydrogen atoms, while the grey sphere in the right image indicates the hydroxide particle in the LMC approach on the oxygen lattice.

The combined Molecular Dynamics/Lattice Monte Carlo approach is implemented in the following stages (cf. Figure 4):

1. AIMD Simulation: A brief *ab initio* molecular dynamics simulation (20 ps) of the aqueous potassium hydroxide solution is conducted to determine the jump rate function ω(*d*_*ij*_) based on Equation 1.
2. Force field MD Simulation: An independent molecular dynamics simulation on nanosecond timescales is performed on pure water, free of ions, to generate the dynamic oxygen lattice. This simulation should be done such that it captures both short‐ and long‐term structural fluctuations (e.g. hydrogen bond network correlations, local density fluctuations) which can be relevant to the diffusion mechanism in water. Instead of bulk water also realistic materials with inhomogenities such as impurities, nanostructure, and confinement can be studied by force field MD. The positions of the oxygen atoms of the water molecules in these system provide the lattice for the Monte Carlo approach. It should be noted that for a specific system (here: a given KOH solution), the oxygen‐oxygen distances have to be further refined via the distance rescaling procedure.
3. Hydroxide Ion Movement: Our Monte Carlo algorithm propagates hydroxide ions across the oxygen lattice using transfer probabilities derived from our calculated jump rate function (first stage). These probabilities are applied to oxygen‐oxygen distances that require careful consideration (second stage). While the initial distances ($d_{O_{\left(H_{2}O\right)}O_{\left(H_{2}O\right)}}$) are extracted from pure water trajectories, we adjust them during the LMC step to accurately apply the appropriate jump rate, accounting for its distance‐dependent behavior. This adjustment is accomplished through our rescaling function, which transforms the standard water‐water distances into hydroxide‐appropriate distances ($d_{O_{\left({\mathrm{OH}}^{-}\right)}O_{\left(H_{2}O\right)}}$) before the jump rate function is applied. This transformation ensures that the structural environment around hydroxide ions is properly represented despite using a water‐based lattice.

The multiscale nature of our approach is evident in the significant computational efficiency achieved: the cost of propagating protons in the Monte Carlo step is reduced by several orders of magnitude compared to the cost of an equivalent AIMD step, enabling simulations at extended timescales with manageable computational resources (**Figure** **4**).

## 3 Results

**Figure 5:**
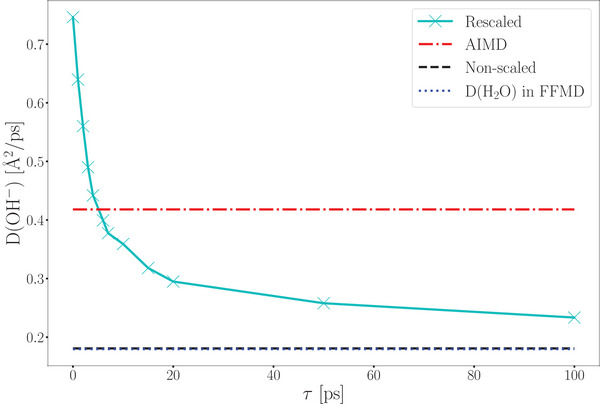
Hydroxide ion diffusion coefficient as a function of relaxation parameter τ. The value τ = 4 ps provides the most accurate alignment with the AIMD simulation.

We applied the proposed cMD/LMC approach to calculate the hydroxide diffusion coefficient, both with and without rescaling the O‐O distances. In the absence of rescaling, the diffusion coefficient of the hydroxide ions is significantly underestimated (0.18  Å^2^ps^−1^) compared to the value obtained from AIMD simulations (0.42 Å^2^ps^−1^) (see **Figure** **5**). The predicted value from the approach without rescaling is comparable to the diffusion coefficient of water molecules observed in both AIMD and force field MD simulations. This is because proton jumps occur very rarely due to the larger distance between two water molecules, *d*_OO_ (unscaled distance values).

Instantaneous rescaling leads to an overestimation of the hydroxide diffusion coefficient (0.75  Å ^2^ ps^−1^), a result also observed in the initial formulation of the multiscale approach for simulating proton dynamics in water.^[^ ^27^ ^]^

To improve the physical accuracy of our framework, we implemented a time‐dependent rescaling approach to supplement the relaxation of the hydrogen bond network following proton transfer. Therefore we require the hydroxide reorientation times measured via femtosecond spectroscopy of the charge transfer to solvent transition of OH^−^ and theoretical studies of the OH^−^ bond reorientation time correlation function, as these are the corresponding real physical phenomena.^[^ ^44^, ^45^, ^46^ ^]^ Using the stated value of τ = 4 ps by Chandra et al.,^[^ ^44^ ^]^ our approach yields a hydroxide diffusion coefficient of *D*_cMD/LMC_(OH^−^) = 0.44  Å^2^ps^−1^, which closely aligns with the diffusion coefficient obtained from the AIMD trajectories (Figure 5). This approach adjusts the rescaling dynamically based on the elapsed time since the jump occurred, governed by the relaxation parameter τ.

To evaluate the diffusion coefficients of the hydroxide ions in the lengthy AIMD simulations (200 ps) experimental values for ionic conductivity were used for comparison.

(2) $$\sigma =\frac{D\cdot q^{2}\cdot c\left(\mathrm{KOH}\right)\cdot N_{A}}{k_{B}\cdot T}$$

The computational ionic conductivities, derived from the AIMD simulations, were calculated using the equation 2 where *q* is the charge of the moving ion. For monovalent ions, this corresponds to the *elementary charge* (1.602 × 10^−19^ C). *D* represents the diffusion coefficient, *k*_*B*_ is the *Boltzmann constant* (1.381 × 10^−23^ J K^−1^), *T* is the temperature, *N*_*A*_ is the *Avogadro constant* (6.022 × 10^23^  mol^−1^), and *c*(KOH) is the concentration of KOH in the solution.

**Figure 6:**
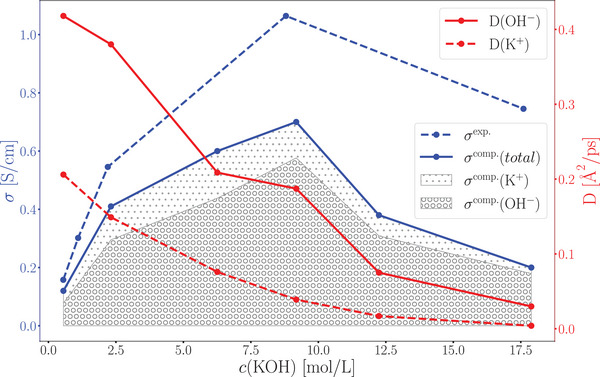
Ionic conductivity σ and diffusion coefficients of OH^−^ and K^+^ ions as a function of KOH concentration at 333 K.

The ionic conductivities computed from the AIMD simulations align with the experimental trend, confirming the reliability of AIMD simulations in capturing local hydroxide transfer rates to use in the cMD/LMC approach. The best agreement between AIMD‐derived and experimental conductivity values is observed for dilute KOH solutions (0.56 molL^−1^: σ^exp.^ = 0.13 Scm^−1^ and σ^comp.^ = 0.12 Scm^−1^) although the agreement slightly deteriorates at higher concentrations (**Figure** **6**).

Since the proton jump behavior in the cMD/LMC approach originates from AIMD, the comparison of *D*_AIMD_(OH^−^) and *D*_cMD/LMC_(OH^−^) remains the primary focus and will be addressed in detail after the next section.

**Figure 7:**
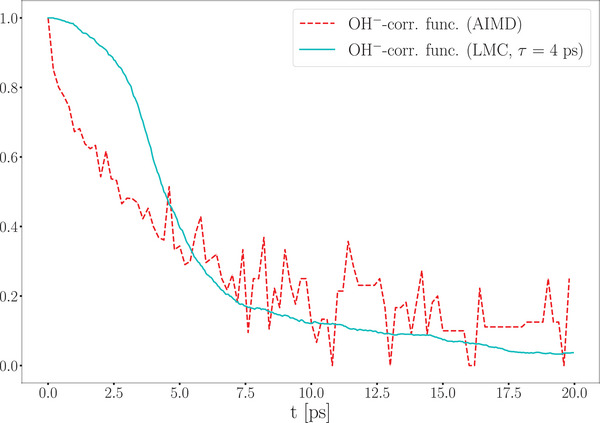
Hydroxide lifetime function obtained from the AIMD trajectory and the cMD/LMC approach.

Another notable outcome of the cMD/LMC approach is the OH^−^ lifetime correlation function, which provides insights into the average lifetime of hydroxide ions. In this context, lifetime refers to the duration of existence of a single protonated oxygen atom. A comparison between AIMD and cMD/LMC results shows that after an initial transient phase — during which the rescaling process in the cMD/LMC approach begins — both methods exhibit highly consistent behavior. The AIMD correlation function decays to zero at approximately 15 ps, while the cMD/LMC correlation function demonstrates a comparable decay profile, underscoring the accuracy of the method (see **Figure** **7**). Furthermore, the half‐life of the AIMD correlation function is 3.5 ps, closely matching the relaxation parameter τ employed in the cMD/LMC framework.

## 4 Predicting Hydroxide Diffusion for Different Concentrations

**Figure 8:**
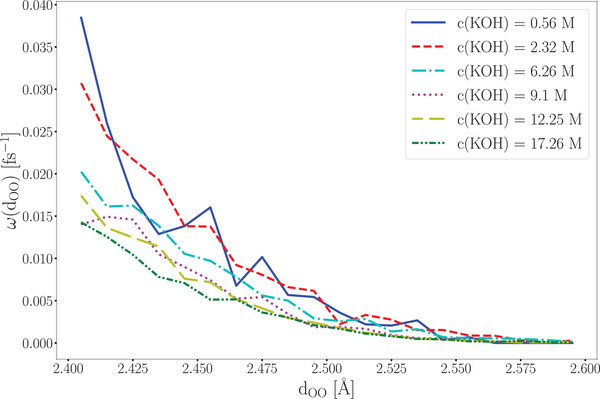
Jump rate functions of the AIMD trajectories with *c*(KOH) = 0.56 molL^−1^ to 17.89 molL^−1^.

The efficiency of the approach is significantly enhanced by enabling the determination of the hydroxide ion diffusion coefficient at various concentrations of potassium hydroxide solutions using only a single short AIMD simulation. A comparison of the jump rate functions obtained from AIMD simulations at different c(KOH) values shows no concentration dependence in the critical range of 2.4 Å to 2.5 Å (see **Figure** **8**). Consequently, the diffusion coefficients of hydroxide ions across different KOH concentrations can be qualitatively estimated with the cMD/LMC approach using a single jump rate function obtained from a short AIMD simulation.

**Figure 9:**
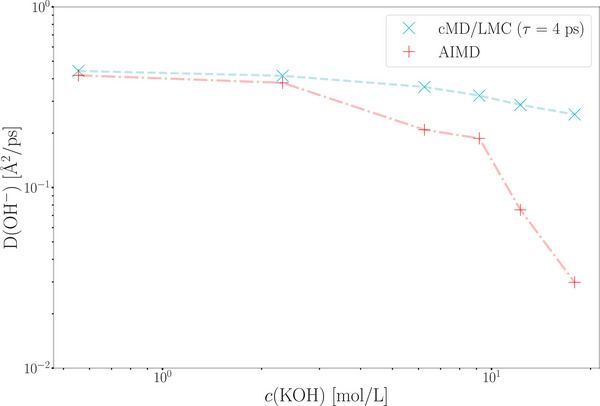
Diffusion coefficients of hydroxide ions at various KOH concentrations, obtained from AIMD simulations and the cMD/LMC method.

**Figure 10:**
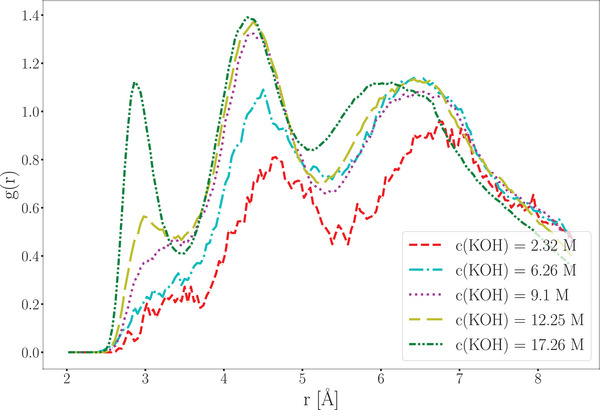
Radial distribution function between the oxygen atoms of two hydroxide moieties.

The hydroxide ion diffusion coefficient at *c*(KOH) = 0.56 molL^−1^ closely matches the value derived from AIMD simulations. At *c*(KOH) = 2.32 molL^−1^, the cMD/LMC method provides a reasonably accurate prediction of the diffusion coefficient (see **Figure** **9**). However, at higher KOH concentrations, the method's accuracy decreases due to increased ion interactions, which hinder diffusion, a trend that is reflected in the radial distribution function of the oxygen atoms of the hydroxide ions (see Figures 9 and **10**). At potassium hydroxide concentrations of c(KOH) ⩾ 12.25 molL^−1^, a distinct first peak appears in the RDF, at a short distance of approximately 2.9 Å (see Figure 10). This feature is absent at lower concentrations. At these elevated concentrations, hydroxide ions displace water molecules from the solvation shells of other hydroxide ions, resulting in a separation of approximately 2.9 Å between hydroxide oxygen atoms. This proximity enables the formation of weak hydrogen bonds despite the repulsive electrostatic interactions.

Despite this limitation, the method remains effective for capturing the general trend of hydroxide ion diffusion across a range of concentrations. As the KOH concentration increases, the viscosity of the solution also increases. To account for this, we adjusted the viscosity according to experimental values by changing the temperature in the simulation. Details of the modified force field MD simulations are provided in the computational details section.

We illustrate our method using temperature variation as a concrete external perturbation that alters the oxygen–atom lattice. By running pure water MD at different temperatures, we are able to mimic the increased viscosity ‐ and consequent slowing of oxygen lattice dynamics ‐ observed at higher solute concentrations. More generally, our approach can also be used to simulate other perturbations arising from material heterogeneities that affect the oxygen positions and dynamics, such as chemical impurities (e.g., gas bubbles), nanoscale structuring, and confinement effects.

### 4.1 Reduction of the Number of Empirical Parameters

In this section, we demonstrate that our approach can be applied without the need for empirical parameters by using hydroxide solution trajectories instead of pure water. This simplification enables application without the need for distance rescaling according to the relaxation time τ or temperature adjustment for viscosity.

Recently, de Lucas *et al.* ^[^ ^18^ ^]^ fine‐tuned a non‐reactive force field designed specifically to describe OH^−^ ions in water more accurately within the TIP4P model. This parameterization accurately reproduces many descriptors of alkaline solutions ‐ particularly radial distribution functions and viscosities ‐ but cannot directly simulate hydroxide diffusion, since bond breaking and formation in the Grotthuss mechanism are absent. However, running classical MD with this force field at different concentrations immediately eliminates the need to correct viscosity via temperature changes.

**Figure 11:**
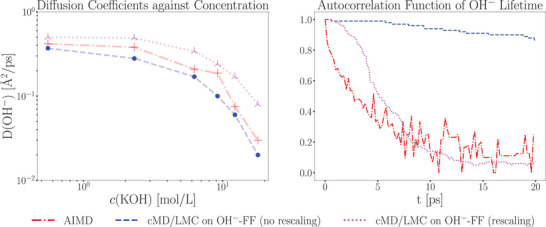
Diffusion coefficients and autocorrelation functions for the approach with and without rescaling of the cMD/LMC on hydroxide ion containing FFMDs against the AIMD data.

Furthermore, the need for distance rescaling is reduced, because the explicitly treated hydroxide ions in the classical MD already form closer contacts to water molecules than the typical water–water separation. In **Figure** **11** we report LMC–predicted diffusion coefficients without any rescaling (i.e., with no relaxation parameter τ). This simplified framework qualitatively captures the concentration dependence but systematically underestimates the AIMD reference values, as the LMC algorithm follows paths defined by the force field MD and only sporadically superimposes proton–transfer events. When we apply the rescaling procedure to trajectories containing explicit hydroxide ions, we again reproduce the concentration–dependence trend ‐ albeit with a systematic overestimation (left panel). Crucially, only the rescaled framework correctly reproduces the OH^−^ lifetime correlation function behavior (right panel of Figure 11).

Our approach can predict hydroxide dynamics across various concentrations without the need for temperature rescaling. It also reproduces the diffusion coefficient ‐ though not the OH^−^ lifetime correlation function ‐ in a parameter‐free framework. This highlights the importance of incorporating proton jumps to emulate the Grotthuss mechanism. Consequently, rescaling the oxygen–oxygen distance becomes essential for accurately simulating hydroxide mobility on top of a molecular dynamics trajectory of neutral water. In the approach of Dutta et al., the coordination geometry of the hydroxide ion's first solvation shell is accounted for by interleaving force field MD simulation steps between Monte Carlo proton‐hopping steps, using a parametrized TIP3P water model that explicitly includes hydroxide ions.

## 5 Outlook

Another study, published in December 2024, focuses on predicting the diffusion of hydroxide ions in an aqueous environment using a similar method.^[^ ^47^ ^]^ This approach is also based on FFMD simulations combined with a mechanism to model proton jumps. Unlike our method, the model proposed by Dutta et al. alternates between Monte Carlo and molecular dynamics steps, allowing proton jumps to influence the subsequent trajectory of the oxygen atoms.

In contrast, our approach involves generating the water trajectory using FFMD simulations and then propagating protons on the lattice of the oxygen atoms via a Monte Carlo method. Furthermore, unlike Dutta et al., the jump probabilities in our Monte Carlo steps are derived from a distance‐dependent jump rate function, which we determine through a short AIMD simulation of aqueous hydroxide solution. The method described in the aforementioned study utilizes a modified Metropolis criterion with an empirical threshold parameter, calibrated to achieve the correct diffusion coefficient. By applying the MD–Monte Carlo technique to simulate ion transfer in the system, the determination of OH^−^ diffusion coefficients remains physically interpretable, rather than relying on the black‐box nature of machine‐learning‐based interatomic potentials. Moreover, this approach is faster than classical force field molecular dynamics, while machine‐learning molecular dynamics are comparatively slower, making the LMC method particularly well‐suited for investigating ion dynamics over extended timescales and within complex environments.

Since our method relies on a pre‐generated water trajectory (without hydroxide ions), we employ a distance‐rescaling mechanism and a relaxation parameter to accurately represent the hydrogen‐bond network dynamics in the presence of hydroxide ions. Future research will combine the method of Dutta et al. with our approach. Such a hybrid method might involve alternating MD and Monte Carlo steps (as in the approach of Dutta et al.) while utilizing a distance‐dependent jump rate function, sampled from AIMD simulations, to calculate proton transfer probabilities (as in our approach).

Future work could extend our framework to investigate temperature‐dependent reorientation times of hydroxide ions in aqueous solutions.^[^ ^46^ ^]^ By sampling jump rate functions at various temperatures from AIMD simulations and incorporating them into our statistical framework, we could potentially reproduce experimental trends reported in spectroscopic studies.

## 6 Conclusion 

Building on the computationally demanding AIMD method, which accurately simulates atomistic processes such as covalent bond formation and breakage (including proton diffusion via the Grotthuss mechanism) but is limited to a few hundred atoms over a few picoseconds, we adapted and applied a combined Molecular Dynamics/Lattice Monte Carlo approach. This method enables the modeling of hydroxide ion transfer over significantly larger timescales and extended system sizes. The proton transfer algorithm was successfully applied to a range of concentrations of aqueous KOH solutions. The cMD/LMC approach delivers reliable results for KOH solutions at low concentrations.

However, at exceptionally high concentrations, the method's accuracy decreases due to 1) the elevated viscosity of the solutions, which is only incorporated in our simulation scheme by reducing the temperature in the underlying force field MD simulations and 2) the unusual formation of hydrogen bonds between hydroxid ions at very high hydroxide concentrations.

The empirical relaxation parameter τ, used to describe the hydrogen‐bond network's response time to a proton jump, was set to 4 ps. This value aligns closely with the mean time between proton jumps in the AIMD simulation (3 ps) and the half‐life of the hydroxide lifetime correlation function (3.5 ps).

The cMD/LMC approach provides a computationally efficient framework for simulating aqueous KOH solutions, covering timescales from nanoseconds to milliseconds and systems involving several thousand atoms. This method enables the detailed characterization of OH^−^ dynamics in complex nanostructered systems, including anion‐exchange membranes and layered double hydroxides, offering a valuable tool for advancing research in these areas.

## 7 Computational Details

### 7.1 Ab Initio Molecular Dynamics Simulation of KOH in Aqueous Solution

Systems with KOH concentrations from 0.56 molL^−1^ to 17.89 molL^−1^ were simulated. Their details are listed in **Table** **2**.

**Table 2:** Computational details of the AIMD simulations.

simulation of	1 KOH	4 KOH	10 KOH
	in 98 H_2_O	in 92 H_2_O	in 80 H_2_O
*c*(KOH) [molL^−1^]	0.56	2.32	6.26
w(KOH) [%]	3	12	28
box size:			
*x* [Å]	14.41	14.21	13.85
*y* [Å]	14.41	14.21	13.85
*z* [Å]	14.41	14.21	13.85
angle [°]	α = 90	β = 90	γ = 90
number of atoms	297	288	270
duration of time step [fs]	0.5	0.5	0.5
temperature [K]	333	333	333
simulation time [ps]	200	200	200
energy drift [Hafs^−1^]	4.7 · 10^−9^	9.8 · 10^−8^	1.6 · 10^−7^

simulation of	14 KOH	18 KOH	25 KOH
	in 72 H_2_O	in 64 H_2_O	in 50 H_2_O
*c*(KOH) [molL^−1^]	9.18	12.25	17.89
w(KOH) [%]	37	48	61
box size:			
*x* [Å]	13.64	13.46	13.24
*y* [Å]	13.64	13.46	13.24
*z* [Å]	13.64	13.46	13.24
angle [°]	α = 90	β = 90	γ = 90
number of atoms	258	246	225
duration of time step [fs]	0.5	0.5	0.5
temperature [K]	333	333	333
simulation time [ps]	250	250	350
energy drift [Ha fs^−1^]	5.7 · 10^−8^	1.1 · 10^−7^	1.1 · 10^−8^

The structures were subjected to a geometry optimization before simulation. The software package CP2K^[^ ^48^, ^49^, ^50^ ^]^ for quantum chemistry and solid state physics was used for this purpose as well as for the *ab initio* molecular dynamic simulations that were performed at temperatures of 333 K. The trajectories comprise 200 ps with a timestep every 0.5 fs.

The electronic structure was modeled with these quantum chemical calculations utilizing the density‐functional theory (DFT).^[^ ^51^, ^52^, ^53^ ^]^ The module Quickstep^[^ ^54^ ^]^ and an efficient orbital transformation method^[^ ^55^ ^]^ were chosen in favor of a fast convergence. The BLYP‐functional was used as the XC‐functional.^[^ ^56^, ^57^ ^]^ Moreover a basis set of the type DZVP‐MOLOPT‐SR‐GTH,^[^ ^58^ ^]^ GTH‐BLYP pseudo‐potentials^[^ ^59^, ^60^, ^61^ ^]^ and the empirical dispersion correction form Grimme (D3)^[^ ^62^, ^63^ ^]^ were applied. The jumps which are part of the long‐range proton transfer from these simulations are defined to be real jumps.

### 7.2 Force Field Molecular Dynamics Simulation of Water

The classical molecular dynamics simulations were obtained with the “Large‐scale Atomic/Molecular Massively Parallel Simulator” (LAMMPS) utilizing the TIP4P water model.^[^ ^64^, ^65^ ^]^ Their details are listed in **Table** **3**. The temperature of the FFMD simulation was adjusted to ensure that the diffusion coefficients of water molecules in FFMD matched the values obtained from AIMD at higher concentrations.

**Table 3:** Computational details of the FFMD simulations.

simulation of	256 H_2_O	256 H_2_O	256 H_2_O	256 H_2_O	256 H_2_O	256 H_2_O
emulating w(KOH) [%]	3	12	28	37	48	61
temperature [K]	288	278	268	253	243	228
box size:						
*x* [Å]	19.71	19.71	19.71	19.71	19.71	19.71
*y* [Å]	19.71	19.71	19.71	19.71	19.71	19.71
*z* [Å]	19.71	19.71	19.71	19.71	19.71	19.71
angle [°]	α = 90;β = 90; γ = 90					
number of atoms	768	768	768	768	768	768
duration of time step [fs]	0.5	0.5	0.5	0.5	0.5	0.5
simulation time [ps]	2500	2500	2500	2500	2500	2500

## 8 Experimental Details

**Figure 12:**
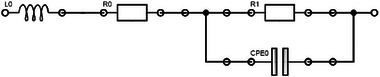
Equivalent circuit for fitting EIS measurements consisting of an inductor, two ohmic resistors and a constant phase element (CPE).

The conductivity of the KOH solution was measured via electrochemical impedance spectroscopy (EIS) with a potentiostat (Zahner^®^ Zennium Pro) connected to an in‐house made cell that was heated in an oven. Two symmetrical round metal plates made from nickel (Ni 2.4060) were used as electrodes. The surface area wetted by the electrolyte was 18.2  cm^2^  and the distance between the electrodes was 3.17 cm. The electrolyte was freshly prepared from KOH pellets (⩾ 85.0 %) with Millipore water (18.2 MΩcm at 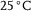). EIS was carried out potentiostatic at 100 mV with an amplitude of 10 mV and a frequency range of 1 to 300000 Hz. 4 steps per decade and 4 measure periods were chosen below 66 Hz and 10 steps per decade and 20 measure periods above 66 Hz. The measurement result was fitted to an equivalent circuit (see **Figure** **12**) consisting of an inductor, two ohmic resistors and a constant phase element (CPE).

*R*_0_ was used to calculate the conductivity of the KOH solution σ^exp.^ with Equation 3. *A* is the surface area wetted by the electrolyte and *l* is the distance between the electrodes.

(3) $$\sigma^{\exp.}=R_{0}\frac{A}{l}$$

## Conflict of Interest

The authors declare no conflict of interest.

## Supporting information

Supporting Information

## Data Availability

The data that support the findings of this study are available from the corresponding author upon reasonable request.
