# Supplementary material for: Hydroxide Mobility in Aqueous Systems: Combining Ab Initio Accuracy with Millisecond Timescales
Source: Small. 2025 Jul 17;22(12):2500931. doi: 10.1002/smll.202500931 (PMC12934385; doi:10.1002/smll.202500931)
Supplement: Supplementary file 1 — Supporting Information [file SMLL-22-2500931-s001.pdf]

Supporting Information: Hydroxide Mobility in Aqueous Systems: Combining *Ab Initio* Accuracy with Millisecond Timescales

Jonas Hänseroth,†,‡ Daniel Sebastiani,‡ Johnny Jimenez Siegert,‡ Jakob Scholl,¶ Karl Skadell,¶ and Christian Dreßler*,†

†*Department of Theoretical Solid State Physics, Institute of Physics, Technische Universität Ilmenau, 98693 Ilmenau, Germany*

‡*Theoretical Chemistry, Martin-Luther Universität Halle-Wittenberg, 06120 Halle (Saale), Germany*

¶*Fraunhofer-Institut für Keramische Technologien und Systeme IKTS, Hydrogen Technologies, 99310 Arnstadt, Germany*

E-mail: christian.dressler@tu-ilmenau.de

Supporting Information

Hydroxide dynamics in aqueous systems

The dynamics of hydroxide ions in aqueous solutions have been fundamentally investigated by many in the recent past due to its high mobility compared to other ions. There are several proposed mechanisms describing the proton transport at a molecular level.[1, 2]

The well-known process of the transport of hydrated protons in water, often referred to as structural diffusion or Grotthuss mechanism, is based on fluctuations in their second solvation shell. The coordination number of the H^+ acceptor in the first solvation shell is reduced from four to three by an H-bond break, so that it is suitably prepared for proton transfer. [2] After the O−O distance between the H^+ acceptor and the H^+ donor has been reduced, the H^+ moves to the center of the two O atoms, resulting in an intermediate complex with the formula $H_5O_2{}^+$, also known as Zundel complex. [1] The complete transfer of H^+ leads to a properly solvated H_3O^+ and the fourfold coordination of the former H^+ donor. The transposition of this concept to proton transfer in hydroxide systems is referred to as the proton-hole mechanism which considers OH^- as a water molecule missing a proton. In this picture, a threefold coordinated OH^- and an $H_3O_2{}^-$ intermediate complex analogous to $H_3O^+(H_2O)_3$ and $H_5O_2{}^+$ could be predicted.[3] The transfer would be based on the similar second-solvation-shell fluctuations.[1]

Ab initio studies on the structure and dynamics of hydrated hydroxide however suggest a different mechanism and explain why the mirror image does not apply.[1] The proposed transfer is shown in Figure 3 in the publication from Tuckerman.[1] The first step in the observed proton transport is a transformation from $OH^-(H_2O)_4$ to $OH^-(H_2O)_3$ initiated by an H-bond break between the hydroxide O atom (O*) and the first solvation shell (a to b). During this process, the coordination geometry of the O* changes from square-planar to tetrahedral. It is important to mention that the three lone pairs of the O* have a delocalized ring structure and thus contribute to the hypercoordination of the OH^-. A new, weak H-bond between the hydroxide H atom (H') and a bulk water molecule leads to the activation of the $OH^-(H_2O)_3$ complex (b to c). The H-bond donated by the H' is necessary for the further steps in the transfer process. Its existence can be proven using the Fourier transform infrared (FTIR) spectroscopy.[4] Now, a proton of a water molecule of the first solvation shell begins to transfer to the O* (c to d). During the partial proton transfer, the same environment exists for both OH^- moieties. The present complex with the formula $[HO\cdots H\cdots OH]^-$ can

be understood as a Zundel analog. A disturbed ring structure of the electron localization function of the O* can be seen (d). After the proton transfer there is a new hydroxide ion (O*H'), which is threefold coordinated, and the original OH^-, which is transformed into a properly solvated water molecule, located in the first solvation shell of the new OH^- (d to e). The inactivation takes place through the acceptance of a fourth H-bond by the O* (e to f). The hydroxide ion is thus fourfold coordinated and forms a square-planar geometry. The resting state $OH^-(H_2O)_4$ is reached.

In this so called dynamical hypercoordination mechanism, the Zundel analog complex $H_3O_2{}^-$ occurs only as a transient species. This behavior could also be shown by time-resolved IR experiments.[5] The complex is only present for 2 to 3 oscillation periods during the transfer mechanism.[5] In contrast, the traditional proton-hole image predicts that the complex is present as an intermediate and also does not propose a donating H-bond from the hydroxide H atom.[6] The OH^- transfer behavior in aqueous systems demonstrated via the "presolvation concept" can be comprehensively described by the mechanism based on hypercoordination and dynamic solvation shell changes. [1, 6, 7]

In studies, the molecular dynamics simulations based on different density functionals, more precisely PW91, BLYP and HCTH, were compared with each other and the most suitable one was sought.[7] To evaluate the results, the observed diffusion coefficients of water molecules, hydroxide ions and protons were analyzed and compared with experimental values. Tuckerman et al. summarized their findings in Table 1 of their publication.[7] It is well established knowledge that the ion mobility of protons is greater than that of hydroxide ions, both of which, in turn, are also greater than that of water. This fact can only be found looking at the values in the experiment and BLYP columns.[7] In addition to the physically reasonable diffusion coefficients, the authors also considered the properly proposed solvation pattern and the accurate structural diffusion mechanism as justification for using BLYP as the density functional.

Calculation of the hydroxide lifetime function

The lifetime function of the hydroxide moieties is calculated using the dot product of two one-dimensional arrays with the length of the number of oxygen atoms in the system. The elements of the arrays are 0 if the corresponding O atom is part of a water molecule and 1 in the case of a hydroxide O atom. The dot product of the array at time t of the trajectory and the array at time $t+\tau$ of the trajectory is obtained for all starting times. Equation S1 contains the calculation instructions, T being the total simulation time.

$$f_{\mathrm{OH-lifetime}}(\tau) = N \cdot \langle \sum_{t=0}^{T-\tau} A_i(t) \cdot A_i(t+\tau) \rangle_i \tag{S1}$$

Concentration dependency of the jump rate function

The jump rate functions exhibits no concentration dependency (see Fig. S1). It is important to note that the function derived from the $0.56\,\mathrm{mol\,L^{-1}}$ KOH solution is based on a significantly smaller number of observed proton jumps. Additionally, at short oxygen-oxygen distances (2.35 and below), jumps are rare, and the frequency of these distances in the trajectory is very low. This results in poor statistical reliability and introduces artifacts when compared to data from higher concentrations.

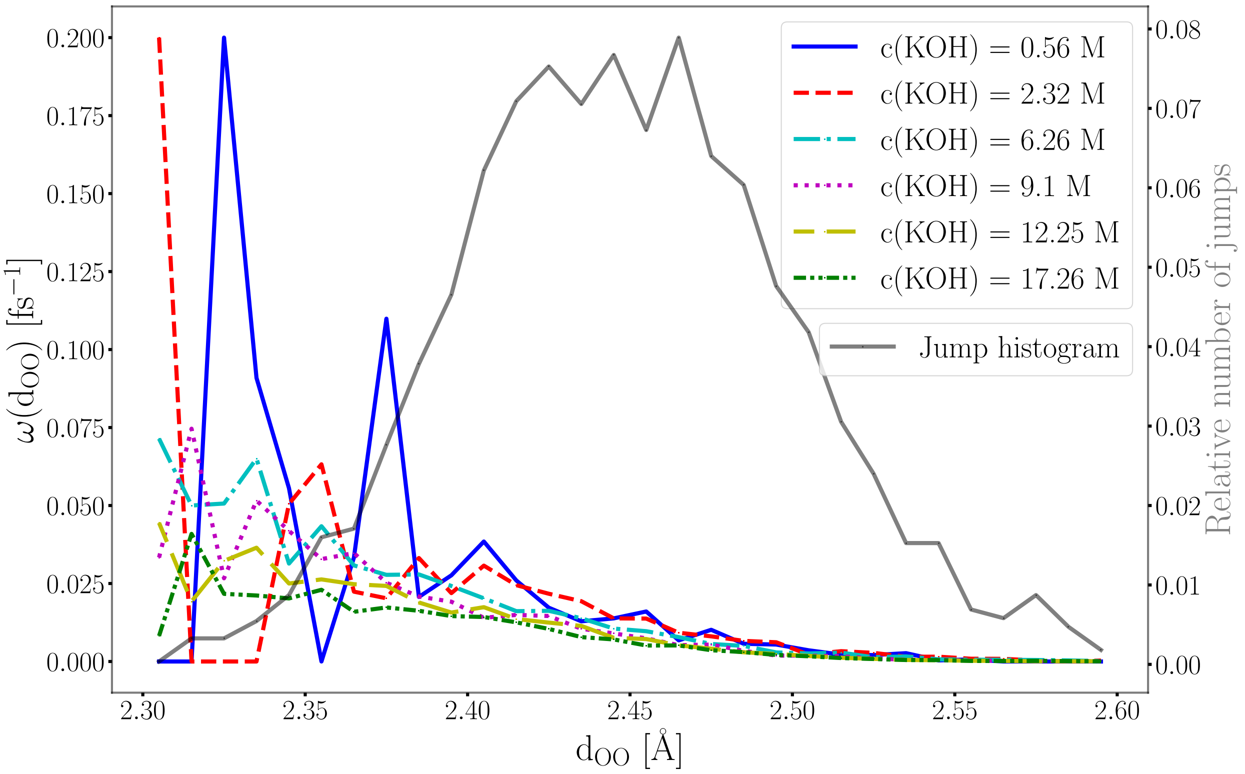

Figure S1: Jump rate functions of the AIMD trajectories with $c(\text{KOH}) = 0.56\,\text{mol}\,\text{L}^{-1}$ to $17.89\,\text{mol}\,\text{L}^{-1}$ from 2.3 to 2.6 with the normalized jump histogram.

References

[1] M. Tuckerman, D. Marx, and M. Parrinello. "The nature and transport mechanism of hydrated hydroxide ions in aqueous solution". In: *Nature* 417.6892 (2002), pp. 925–929.

[2] M. Tuckerman, K. Laasonen, M. Sprik, and M. Parrinello. "Ab Initio Molecular Dynamics Simulation of the Solvation and Transport of H3O+ and OH- Ions in Water". In: *The Journal of Physical Chemistry* 99.16 (1995), pp. 5749–5752. DOI: 10.1021/j100016a003.

[3] G. Zatsepina. "State of the hydroxide ion in water and aqueous solutions". In: *Journal of Structural Chemistry* 12.6 (1972), pp. 894–898.

[4] M. Śmiechowski and J. Stangret. “Hydroxide Ion Hydration in Aqueous Solutions”. In: *The Journal of Physical Chemistry A* 111.15 (2007), pp. 2889–2897. DOI: 10.1021/jp0659397.

[5] S. Roberts, P. Petersen, K. Ramasesha, A. Tokmakoff, I. Ufimtsev, and T. Martinez. “Observation of a Zundel-like transition state during proton transfer in aqueous hydroxide solutions”. In: *Proceedings of the National Academy of Sciences* 106.36 (2009), pp. 15154–15159. DOI: 10.1073/pnas.0901571106.

[6] D. Marx, A. Chandra, and M. Tuckerman. “Aqueous Basic Solutions: Hydroxide Solvation, Structural Diffusion, and Comparison to the Hydrated Proton”. In: *Chemical Reviews* 110.4 (2010), pp. 2174–2216. DOI: 10.1021/cr900233f.

[7] Mark E. Tuckerman, A. Chandra, and D. Marx. “Structure and Dynamics of OH-(aq)”. In: *Accounts of Chemical Research* 39.2 (2006), pp. 151–158. DOI: 10.1021/ar040207n.
